# Intracerebral Haemorrhage During a Caesarean Section: A Report of a Rare Case

**DOI:** 10.7759/cureus.96411

**Published:** 2025-11-09

**Authors:** Megha Gupta, Cesare Bolzonella

**Affiliations:** 1 Obstetrics and Gynaecology, James Cook University Hospital, Middlesbrough, GBR; 2 Obstetrics and Gynaecology, Northampton General Hospital, Northampton, GBR

**Keywords:** caesarean, headache, intraoperative, pregnancy, stroke

## Abstract

We report a rare case of intracerebral haemorrhage (ICH) in a pregnant patient intraoperatively during caesarean section, without a history of hypertension antenatally or an identifiable aetiology on subsequent evaluation. This case underscores the importance of considering atypical causes of stroke in pregnancy and highlights possible predisposing factors for haemorrhagic events in patients without a history of pregnancy-induced hypertension or preeclampsia. We also discuss management of stroke in pregnancy for optimal outcome with a multidisciplinary approach.

## Introduction

Intracerebral haemorrhage (ICH) during pregnancy is associated with high maternal morbidity and mortality. The incidence of stroke in pregnancy is estimated between nine and 26 per 100,000 deliveries, with haemorrhagic stroke accounting for approximately 38% of cases [1]. Most cases occur in the postpartum period, with the relative risk of ICH reported as 2.5 during pregnancy and 28.3 in the puerperium [2].

The leading causes of ICH include vascular malformations such as arteriovenous malformations (AVMs) and aneurysms, accounting for 41% of cases. Other aetiologies include preeclampsia, Moyamoya disease, cavernous angioma, cerebral venous sinus thrombosis (CVST), and intracranial neoplasms. Coagulopathies and infections are less common causes, while a subset of patients remain idiopathic [3].

Pregnancy induces significant hemodynamic and coagulation changes, predisposing to cerebrovascular complications [4]. Hypertensive disorders of pregnancy remain the most important risk factor, implicated in one-third of cases [5].

We describe a rare case of intraoperative haemorrhagic stroke during caesarean section in a patient with no history of hypertensive disease.

## Case presentation

A 30-year-old woman at term gestation underwent an elective caesarean section under spinal anaesthesia. She was a primigravida and had a history of bilateral hip osteoarthritis with right hip replacement. As there was limited mobility at the hip joint, a caesarean section was planned as per the advice of the orthopaedic consultant. The patient had post-traumatic stress disorder from previous theatre experience and suffered from anxiety and depression. She also had a history of anorexia nervosa and was worried about weight gain in pregnancy. She was not on any medication, but due to ongoing mental health issues, she was requested to be first on the elective theatre list. Toward the end of the caesarean section, she developed a sudden, severe headache (pain score 10/10), described as the most severe of her life, associated with neck stiffness. This was preceded by constant retching and vomiting. She had no history of preeclampsia or gestational hypertension. Within minutes, she developed left facial weakness, dysarthria, left-sided visual and sensory neglect, and progressive hemiparesis.

Her past medical history was significant for migraine with aura. She had a family history of preeclampsia and had been on low-dose aspirin 150 mg from 12 weeks of pregnancy until 36 weeks’ gestation. This was to reduce the risk of preeclampsia as the patient had two moderate risk factors for preeclampsia as per the National Institute for Health and Care Excellence (NICE) guidelines. Migraines remained stable during pregnancy and were managed intermittently with sumatriptan. The patient was very anxious at the time of caesarean section. She was known to be under the perinatal mental health team. The patient was a known case of supraventricular tachycardia with a history of ablation done three years ago and was on treatment with bisoprolol 2.5 mg OD. She had been asymptomatic since ablation, apart from a few episodes in this pregnancy. There was no family history of stroke in the patient, but they gave a history of bleeding in the brain in their sister, who underwent a kidney transplant.

On neurological examination, the patient was alert and oriented. Findings included partial gaze palsy, complete hemianopia, weakness of the left lower face, absent motor function in the left upper and lower limbs, and mild to moderate dysarthria without aphasia or ataxia. Her NIH Stroke Scale score was 18, consistent with severe stroke [6].

**Figure 1:**
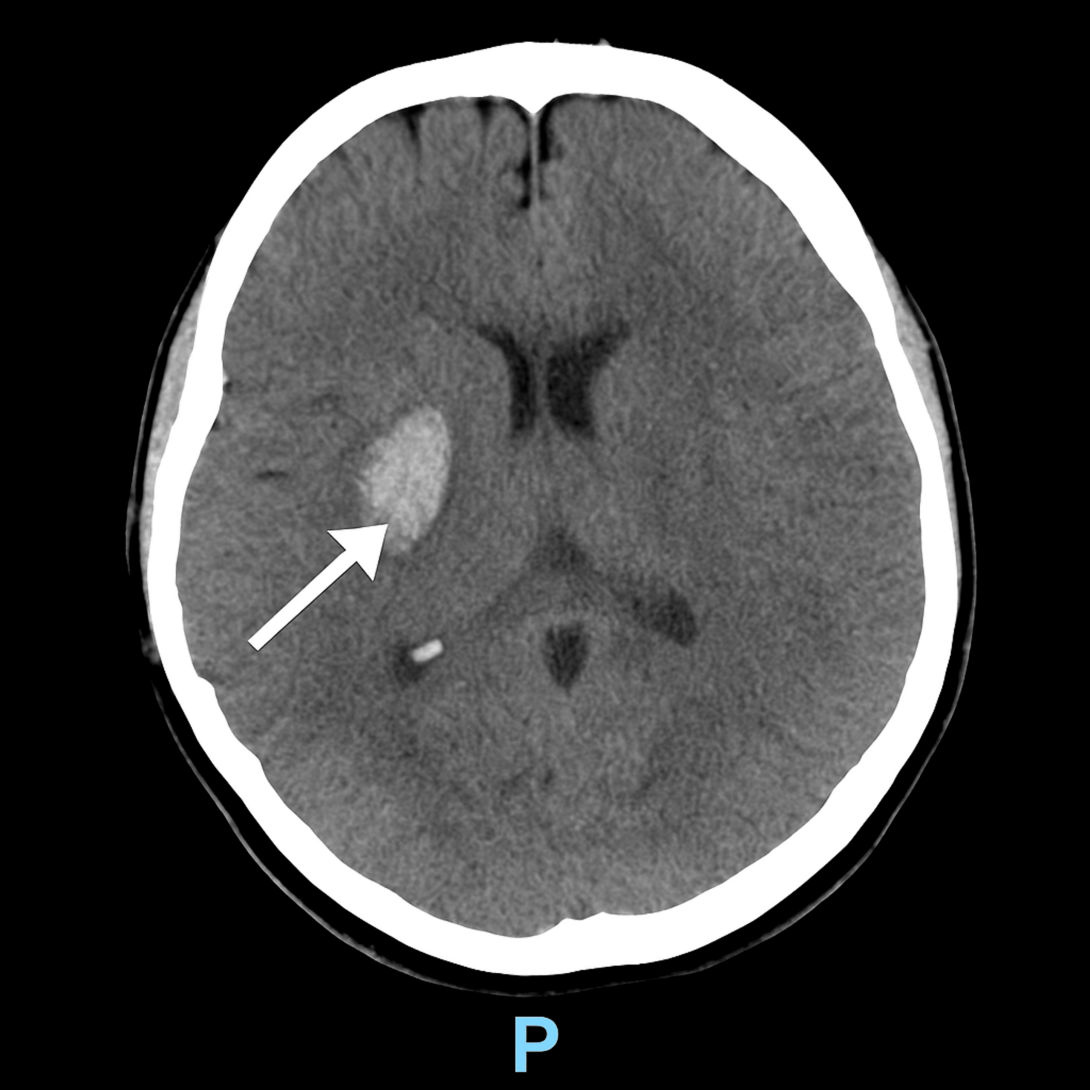
CT scan showing right basal ganglia haematoma

Computed tomography (CT) of the head revealed a right basal ganglia haemorrhage (33 × 26 × 20 mm), reported as “likely hypertensive” (Figure 1). CT angiography demonstrated prominent venous structures in the right M1/M2/M3 territory, raising suspicion of an underlying AVM.

The patient's preoperative anaesthetic assessment was unremarkable, and all investigations were within normal limits. Spinal anaesthesia was uneventful. According to the anaesthetic chart, the patient had two raised BP readings of 145/94mm of Hg and 151/92 mm of Hg. It was clearly documented that the patient had variable dysrhythmia throughout surgery; however, when in sinus rhythm, BP was acceptable. The patient had episodes of sinus rhythm, second-degree heart block, and very short episodes of non-sustained ventricular tachycardia. During these episodes, the BP cuff was struggling to measure, although the radial pulse was well palpable. According to an anaesthetist, the challenge in measuring BP was due to the irregular heart rate rather than hypotension. As per the anaesthetist, it was difficult to determine the cause as BP was normal most of the time.

The patient received ephedrine intraoperatively. Postoperatively, she required metaraminol to maintain systolic BP between 100 and 140 mmHg, attributed to spinal anaesthesia-induced hypotension. Neurosurgical consultation recommended conservative management.

During her intensive care admission, the patient showed spontaneous clinical improvement. Residual deficits included left facial weakness and impaired fine motor function of the left hand. She was transferred to the postnatal ward after five days in ICU and then was discharged two days later for continued care and rehabilitation.

**Figure 2:**
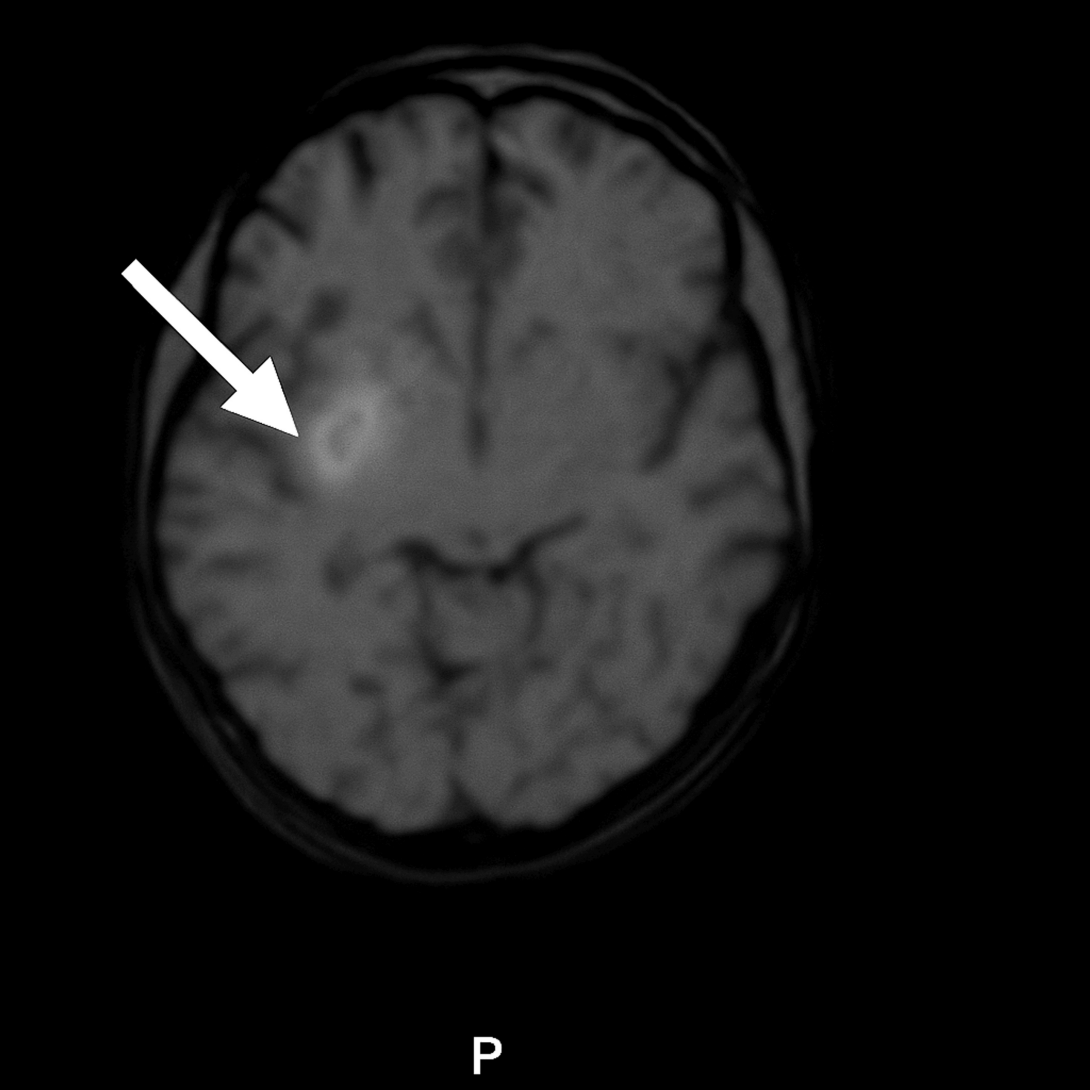
Follow-up MRI showing persistent right basal ganglia haematoma

Follow-up MRI and MRA at four weeks confirmed a persistent right basal ganglia hematoma (Figure 2).

Digital subtraction angiography (DSA) at six weeks excluded AVM and aneurysm. At eight weeks, her neurological function had improved substantially, although mild left-hand weakness persisted.

## Discussion

Stroke in pregnancy is uncommon but associated with severe outcomes. The reported maternal mortality rate is 1.4 per 100,000 deliveries [3]. This case is notable for the occurrence of intraoperative haemorrhagic stroke in the absence of a history of hypertension antenatally, an extremely rare phenomenon with few parallels in the literature.

Preeclampsia and other systemic disorders account for one-third of pregnancy-related strokes, vascular malformations for another third, while the remaining cases are idiopathic [5]. Outcomes are generally more favourable in patients with structural lesions compared to systemic disorders.

Migraine with aura has been recognised as an independent risk factor for stroke. Both ischemic and haemorrhagic strokes are more frequent in women with migraine compared to those without. Women under 45 years with migraine demonstrate a 1.7-2.0-fold increased risk of ischemic stroke [7]. Moreover, headache during pregnancy has been identified as a predictor of postpartum stroke, warranting closer surveillance of such patients [8]. However, in this case, the patient reported no worsening of migraine during pregnancy, and so it is unlikely to be the cause. Moreover, no headache was reported by the patient preoperatively.

Vasopressors used for spinal anaesthesia-induced hypotension may also contribute. Ephedrine, in particular, has been implicated in haemorrhagic stroke during pregnancy, both in clinical reports and in the context of drug abuse [9-12]. According to a case report, the patient developed a severe headache immediately after treatment with a vasopressor following intraoperative spinal-induced hypotension, leading to haemorrhagic stroke [9]. In this case, ephedrine was primarily used, with two documented raised BP readings as mentioned earlier. We believe these sudden changes in blood pressure are unlikely to be causative of stroke. All the recorded BP readings were below 160/110 mm of Hg, which is the accepted threshold for most hypertensive complications. According to the anaesthetic chart, administration of ephedrine did not coincide with the incidence or aggravation of headache. Furthermore, all the BP readings after this and in the postoperative period were normal, making this explanation less likely, though not completely excludable.

Optimal management of stroke in pregnancy requires a multidisciplinary approach. Early neuroimaging is critical: CT remains the most rapid and sensitive tool for acute haemorrhage, MRI is preferred in antenatal and subacute cases, and DSA remains the gold standard for vascular pathology [13]. Anticoagulation is contraindicated in haemorrhagic stroke [14].

According to one published case report, a patient presented on postoperative day 4 following an emergency lower-segment cesarean section with a sudden severe headache and neck stiffness, and computed tomography of the brain demonstrated a subarachnoid haemorrhage. Notably, this patient had no pre-existing medical comorbidities or complications during pregnancy [15]. Similarly, another case report described a patient who developed a severe headache on postoperative day 9 after a cesarean section performed under spinal anaesthesia; neuroimaging revealed an intracranial haemorrhage associated with bilateral occlusion of the internal carotid arteries, leading to a subsequent diagnosis of Moyamoya disease [16]. These cases highlight the importance of heightened vigilance for new-onset headaches in the early postoperative period, particularly when the clinical picture deviates from that of a typical post-dural-puncture headache.

Another report described a patient who underwent an emergency cesarean section for uncontrolled pregnancy-induced hypertension (PIH) and subsequently developed hemiparesis and reduced consciousness on postoperative day 2. CT imaging demonstrated a large ICH, necessitating urgent craniotomy and haematoma evacuation [17]. 

Collectively, these cases underscore the spectrum of postpartum intracranial hemorrhagic events following caesarean section and reinforce the need for prompt neurological imaging in any postpartum woman with severe or atypical headache, regardless of anaesthetic technique or pre-existing risk factors.

In this case report, the patient benefited from timely diagnosis and conservative management, with progressive neurological recovery, although mild deficits persisted at follow-up.

## Conclusions

The incidence of pregnancy-associated stroke is increasing, with significant maternal consequences both physically and emotionally. Prompt recognition and neuroimaging in patients presenting with acute neurological symptoms are essential for optimising outcomes.

This case represents an unusual intraoperative presentation of ICH during caesarean section in a patient with no history of pregnancy-induced hypertension or preeclampsia and with no identifiable aetiology. Careful intraoperative hemodynamic management, cautious use of vasopressors for spinal-induced hypotension, vigilance for neurological symptoms, and recognition of high-risk factors such as migraine may contribute to better prevention and improved outcomes in similar scenarios.

## References

[1] Jaigobin C, Silver FL (2000). Stroke and pregnancy. Stroke.

[2] Dias M, Sekhar L (1990). Intracranial hemorrhage from aneurysms and arteriovenous malformations during pregnancy and the puerperium. Neurosurgery.

[3] Ascanio LC, Maragkos GA, Young BC, Boone MD, Kasper EM (2019). Spontaneous intracranial hemorrhage in pregnancy: a systematic review of the literature. Neurocrit Care.

[4] Miller EC (2019). Preeclampsia and cerebrovascular disease. Hypertension.

[5] Meretoja A, Strbian D, Putaala J (2012). SMASH-U: a proposal for etiologic classification of intracerebral hemorrhage. Stroke.

[6] Lyden P (2017). Using the National Institutes of Health Stroke Scale: a cautionary tale. Stroke.

[7] Kurth T, Slomke MA, Kase CS (2005). Migraine, headache, and the risk of stroke in women: a prospective study. Neurology.

[8] Nam KW, Ha S, Oh MJ (2023). Headaches during pregnancy and the risk of subsequent stroke. J Headache Pain.

[9] Ranasinghe JS, Kafi S, Oppenheimer J, Birnbach DJ (2008). Hemorrhagic stroke following elective cesarean delivery. Int J Obstet Anesth.

[10] Droll KP, Lossing AG (2004). Carotid-jugular arteriovenous fistula: case report of an iatrogenic complication following internal jugular vein catheterization. J Clin Anesth.

[11] Poser CM (1983). Postvaccinal encephalitis. Ann Neurol.

[12] Bruno A, Nolte KB, Chapin J (1993). Stroke associated with ephedrine use. Neurology.

[13] Fairhall JM, Stoodley MA (2009). Intracranial haemorrhage in pregnancy. Obstet Med.

[14] Pessin MS, Estol CJ, Lafranchise F, Caplan LR (1993). Safety of anticoagulation after hemorrhagic infarction. Neurology.

[15] Al-Zubaidi R, Al-Khalidi L, Al-Khayat S (2024). Spontaneous subarachnoid hemorrhage after cesarean section: a case report. J Clin Gynecol Obstet.

[16] Ilyas Y, Öncü K, İlyas K, Beşi R A (2024). Moyamoya disease diagnosed with intracranial hemorrhage after cesarean section under spinal anesthesia: a case report. Cureus.

[17] Matsuda R, Fujimoto T, Tamura K, Motoyama Y, Park YS, Nakase H (2011). Case of postpartum intracerebral hemorrhage due to pregnancy induced hypertension [Article in Japanese]. No Shinkei Geka.

